# 

**Published:** 1956-10

**Authors:** 


### MEDICAL JOURNAL OF THE SOUTH-WEST

*(Incorporating the Bristol Medico-Chirurgical Journal)*

Established 1883

VOL. 71 (iv) October, 1956 NO. 262

### Contents

*PUBLISHED QUARTERLY*

*ANNUAL SUBSCRIPTION* 16/- *POST FREE*

PUBLISHED UNDER THE AUSPICES OF THE
BRISTOL MEDICO-CHIRURGICAL SOCIETY

### *Editorial Committee*

Dr. J. E. Cates, Department of Medicine, Bristol Royal Infirmary. *Editor.*
Dr. N. J. Brown, Southmead Hospital, Bristol. *Assistant Editor.*
Dr. J. Macrae, Ham Green Hospital.
Dr. R. C. Wofinden, Central Clinic, Bristol.
Mr. A. E. S. Roberts, Medical Library, University of Bristol. *Secretary.*

### *Local Correspondents*

*Bath* . . Mr. A. D. Bateman, 3 The Circus, Bath.
*Exeter* . . Dr. L. N. Jackson, Mount Jocelyn, Crediton, Devon.
*Gloucester* . Dr. R. F. Jarrett, 9 College Green, Gloucester.
*Plymouth* . Dr. C. R. Croft, Lexden, Hartley Av., Plymouth.
*Taunton* . Dr. M. McAlley, 1 The Crescent, Taunton.
*Torquay* . Dr. A. Robinson Thomas, 20 Devon Square, Newton Abbot, Devon.
*Truro* . . Dr. F. D. M. Hocking, St. Andrews, Perranporth, Cornwall.
*Yeovil* . . Mr. J. A. Vere Nicoll, Knapp House, Yeovil, Somerset.

### NOTICE TO CONTRIBUTORS

Original articles are invited, provided they have not been published elsewhere. Notes of forthcoming events, meetings held, degrees conferred, staff appointments, and other local news are welcomed. The editorial committee reserves the right to make literary corrections.

Illustrations should always be supplied ready for reproduction. All re-touchings or other operations required will be charged to the author. Line drawings should be drawn in Indian ink. We regret that we must ask authors to pay for making blocks, if this is necessary.

J. W. ARROWSMITH LTD., WINTERSTOKE ROAD,
BRISTOL.

### *Notes and News*

Dr. R. Woolmer gave the Frederic Hewitt Lecture on Artistry and Science in Anaesthesia at the annual meeting of the Association of Anaesthetists of Great Britain and Ireland, held at the Royal College of Surgeons of England 1st and 2nd November, 1956.

Mr. G. J. Hadfield has been appointed Senior Lecturer in Surgery in the University of London at St. Bartholomew's Hospital.

*Honorary Physician to the Queen for* 1956–59

Professor I. G. Davies.

Dr. G. C. Kelly.

### *Appointments*

### *UNITED BRISTOL HOSPITALS*

*June to October* 1956

| *Name* | *Appointment* | *Formerly* |
|---|---|---|
| McCormick, A. J. A., M.B., F.R.C.S., D.O.M.S. | Senior Registrar in Ophthalmology. | Registrar, U.B.H. |
| Heaton, J. M., M.B., B.Chir., D.O. | Registrar in Ophthalmology. | Registrar, Oxford Eye Hospital. |
| Eve, N. H., M.B., B.S., D.A. | Registrar in Anaesthetics. | S.H.O., U.B.H. |
| Clarke, A. D., M.B., Ch.B., F.F.A.R.C.S., D.A. | Registrar in Anaesthetics. | S.H.O., Southmead Hospital. |
| Isaacson, A. H., M.A., M.D., M.R.C.P., B.Ch. | Registrar in Radiodiagnosis. | Part-time. |
| Doyle, F. H., B.Sc., M.B., Ch.B. | Registrar in Radiodiagnosis. | Part-time. |
| Patton, Mrs. Irmgard, M.D. (Greifswald), L.A.H. (Dublin). | Registrar in Radiodiagnosis. | H.P., Gulson Hospital. |
| Nobbs, Mrs. Marjorie, B.Sc., M.B., Ch.B., D.M.R.T. | Registrar in Radiotherapy. | S.H.O., U.B.H. |
| Lawrence, A. C. K., M.B., B.S. | Registrar in Pathology. | R.A.F. Institute of Pathology, Halton. |
| Ayliffe, G. A. J., B.Sc., M.B., Ch.B. | Registrar in Pathology. | S.H.O. in Pathology, U.B.H. |
| Mawer, Miss Joan, M.B., B.S., D.C.H. | Registrar in Paediatrics. | Registrar, South Devon and East Cornwall Hospital, Plymouth. |
| Cameron, I. W., L.D.S., B.D.S. | Registrar in Dental Surgery. | S.H.O., U.B.H. |
| Irwin, J. A. L., B.D.Sc., L.D.S. (Melbourne). | Registrar in Orthodontics. | North West Metropolitan Regional Hospital Board. |

### *SOUTH-WESTERN REGIONAL HOSPITAL BOARD*

*June to October* 1956

### *BRISTOL*

| *Name* | *Appointment* | *Formerly* |
|---|---|---|
| Mather, H. G. M.D. (Western Reserve), M.A., M.B., B.Chir.(Camb.), M.R.C.P. (London), M.D. (Camb.). | Consultant Physician, Southmead Hospital. | Senior Medical Registrar—joint appointment with S.E. Metropolitan R.H.B. and King's College Hospital. |
| Pelmore, J. F., M.R.C.S., L.R.C.P., D.A. (R.C.P. & S.), F.F.A.R.C.S. | Consultant Anaesthetist, Frenchay Hospital. | Consultant Anaesthetist, Tunbridge Wells Group of Hospitals. |
| Lyon, H. F., M.B., Ch.B. (Edin.), D.T.M. (Antwerp), M.R.C.P. (Edin.). | Assistant Physician (Geriatrics), Manor Park Hospital, Bristol. | Medical Registrar, Falkirk Royal Infirmary. |
| Burns, Margaret E., M.B., Ch.B. (B'ham), D.C.H., D.A. | Anaesthetic Registrar, Southmead Hospital. | Registrar, Dudley Road Hospital, Birmingham. |

| *Name* | *Appointment* | *Formerly* |
|---|---|---|
| GUY, JOAN, B.SC. (WALES), M.B., B.CH. (WALES), D.C.H. | Registrar to the South-West Regional Blood Transfusion Centre, Southmead. | Registrar in Pathology, United Bristol Hospitals. |
| VALAES, T., M.B. (ATHENS), D.C.H. (ENGLAND). | Paediatric Registrar, Southmead Hospital. | Casualty Officer, Paddington Green Children's Hospital. |
| GREGORY, C., M.B., CH.B. (LIVERPOOL). | Registrar in Psychiatry, Bristol Mental Hospitals. | Asst. Lecturer in Physiology, University of Liverpool. |
| GRANT, BRENDA, M.B., CH.B. (LIVERPOOL), D.P.M. (ENGLAND) & R.C.P.S.I.). | Registrar in Psychiatry, Bristol Mental Hospitals. | S.H.O. in Psychiatry, Bristol Mental Hospitals. |
| WARNER, J., M.B., CH.B. (B'HAM). | Registrar in Child Psychiatry and Mental Deficiency, Bristol. | Registrar in Psychiatry, Bristol Mental Hospitals. |

### *NORTH GLOUCESTERSHIRE*

| | | |
|---|---|---|
| DOWNING, W. B. L., M.B., CH.B. (B'HAM), F.R.C.S. (EDIN.), D.L.O. (R.C.S.). | Consultant Ear, Nose and Throat Surgeon, North Gloucestershire Clinical Area. | Senior Registrar, United Birmingham Hospitals. |
| TOOMEY, A. G. G., B.SC., M.R.C.S., L.R.C.P., D.M.R.D. | Consultant Radiologist, North Gloucestershire Clinical Area. | Senior Registrar, North Gloucestershire Clinical Area. |
| WYPKEMA, W., M.B., CH.B. (PRETORIA). | Surgical Registrar, Cheltenham General Hospital. | S.H.O. in General Surgery, United Bristol Hospitals. |
| STALLMAN, J. F. H., M.B., B.S. (LOND.), F.R.C.S. (ENG.). | Clinical Assistant in Orthopaedic and Traumatic Surgery, North Gloucestershire Clinical Area. | Retired Consultant in Orthopaedic and Traumatic Surgery, North Gloucestershire Clinical Area. |

### *BATH*

| | | |
|---|---|---|
| BOLLEN, A. R., M.B., CH.B. (BRISTOL). | Anaesthetic Registrar, Bath Group of Hospitals. | S.H.O., Sheffield Royal Hospital. |
| BAZELEY, R. W., M.B., B.S. (LONDON). | Appointed for one year as Clinical Assistant in General Medicine to undertake one weekly session in the Bath Group of Hospitals. | In general practice, Radstock. |
| BURROWES, W. L., M.D., B.CH. (BELFAST), M.R.C.P.I. | Re-appointed for further year as Clinical Assistant in General Medicine to undertake one weekly session in the Bath Group of Hospitals. | In general practice, Corsham. |
| ELLISON, D. J., M.B., CH.B. (EDIN.), M.R.C.P. (EDIN.). | Ditto | In general practice, Melksham. |
| RONN, H. H., M.B., B.S. (LONDON), M.R.C.P. (LONDON). | Appointed for one year as Clinical Assistant in General Medicine to undertake one weekly session in the Bath Group of Hospitals. | In general practice, Westbury. |
| WALT, A. J., M.B., CH.B. (CAPE TOWN), F.R.C.S. (C.), M.S. (MINNESOTA). | Surgical Registrar, Bath Group of Hospitals. | Fellow in Surgery, The Mayo Foundation, Rochester, Minnesota, U.S.A. |
| MACMATH, I. F., M.B., CH.B. (GLASGOW), B.SC., D. (OBST.), R.C.O.G. | Clinical Assistant in Obstetrics and Gynaecology at Trowbridge and Bradford-on-Avon Hospitals (1 session). | Supervising Officer, Bradford-on-Avon Maternity Hospital and Trowbridge District Hospital. |
| RIVETT, P. A. H., M.B., CH.B. (LEEDS), M.D., D.C.H. | Re-appointed for further year as Clinical Assistant in Paediatrics, Royal United Hospital (1 session). | In general practice, Calne. |

### DEVON AND CORNWALL

| *Name* | *Appointment* | *Formerly* |
|---|---|---|
| THORNE, M. G., B.A. (CANTAB), M.B., B.CHIR., M.R.C.P. (LONDON), M.D. (CANTAB). | Consultant Physician, Torbay Hospital, Torquay. | Tutor in Medicine to the Pre-Clinical Class, Guy's Hospital. |
| MACMAHON, B. O. P., M.B., B.CH., B.A.O. (N.U.I.), M.D. (N.U.I.), M.R.C.P.I. | Senior Medical Registrar, West Cornwall Clinical Area. | Registrar, St. Kevin's Hospital, Dublin. |
| DONNISON, A. B., M.R.C.S., L.R.C.P., M.B., B.CH. (CANTAB), M.A. (CANTAB), D.C.H. | Paediatric Registrar, South Devon and East Cornwall Hospital, Plymouth (joint appointment with the United Bristol Hospitals). | Registrar, Tadworth Court, Country Branch of Hospital for Sick Children, Great Ormond Street. |
| PARKES, J., M.A., M.B., B.CHIR., M.R.C.P. | Re-appointed for further year as Clinical Assistant in Paediatrics, Torbay Hospital and Rosehill Children's Hospital, Torquay. | In general practice, Torquay. |
| KEMP, MARY, M.R.C.S., L.R.C.P. | Clinical Assistant in Radiotherapy, Royal Devon and Exeter Hospital, Exeter. | — |

